# Recombinant Rod Domain of Vimentin Reduces SARS-CoV-2 Viral Replication by Blocking Spike Protein–ACE2 Interactions

**DOI:** 10.3390/ijms25052477

**Published:** 2024-02-20

**Authors:** Fong Wilson Lam, Cameron August Brown, Shannon Elizabeth Ronca

**Affiliations:** 1 Department of Pediatrics, Baylor College of Medicine, Houston, TX 77030, USAronca@bcm.edu (S.E.R.); 2 Center for Translational Research on Inflammatory Diseases, Michael E. DeBakey Veterans Affairs Medical Center, Houston, TX 77030, USA; 3 Department of Pathology, Baylor College of Medicine, Houston, TX 77030, USA; 4 William T. Shearer Center for Human Immunobiology, Baylor College of Medicine and Texas Children’s Hospital, Houston, TX 77030, USA

**Keywords:** SARS-CoV-2, COVID-19, vimentin, acute lung injury

## Abstract

Although the SARS-CoV-2 vaccination is the primary preventive intervention, there are still few antiviral therapies available, with current drugs decreasing viral replication once the virus is intracellular. Adding novel drugs to target additional points in the viral life cycle is paramount in preventing future pandemics. The purpose of this study was to create and test a novel protein to decrease SARS-CoV-2 replication. We created the recombinant rod domain of vimentin (rhRod) in *E. coli* and used biolayer interferometry to measure its affinity to the SARS-CoV-2 S1S2 spike protein and the ability to block the SARS-CoV-2–ACE2 interaction. We performed plaque assays to measure rhRod’s effect on SARS-CoV-2 replication in Vero E6 cells. Finally, we measured lung inflammation in SARS-CoV-2-exposed K18-hACE transgenic mice given intranasal and intraperitoneal rhRod. We found that rhRod has a high affinity for the S1S2 protein with a strong ability to block S1S2–ACE2 interactions. The daily addition of rhRod decreased viral replication in Vero E6 cells starting at 48 h at concentrations >1 µM. Finally, SARS-CoV-2-infected mice receiving rhRod had decreased lung inflammation compared to mock-treated animals. Based on our data, rhRod decreases SARS-CoV-2 replication in vitro and lung inflammation in vivo. Future studies will need to evaluate the protective effects of rhRod against additional viral variants and identify the optimal dosing scheme that both prevents viral replication and host lung injury.

## 1. Introduction

The coronavirus disease 2019 (COVID-19) pandemic fundamentally changed the world [1,2,3,4]. COVID-19, caused by severe acute respiratory syndrome coronavirus-2 (SARS-CoV-2), first appeared in Wuhan, China, and quickly spread globally. SARS-CoV-2 causes a respiratory infection that spreads through droplets and can lead to devastating long-term outcomes. The severity of the disease has changed throughout the course of the pandemic, with decreasing mortality over time [5,6]. Globally, the infection fatality rate is estimated at ~1% [7] and ~2.5% in the US [8]. In addition to the pulmonary disease, patients infected with SARS-CoV-2 may also develop neurologic, renal, and cardiac complications [9]. These complications may be due to the pathologic activation of the coagulation [10,11,12] and inflammatory systems [13,14]; therefore, preventing infection through vaccination as well as limiting viral replication once infected are paramount to stop the spread of disease.

One tactic to mitigate infection is to prevent viral attachment onto host cells. Current data suggest that SARS-CoV-2 uses its spike protein to bind to the angiotensin-converting enzyme-2 receptor (ACE2) [15,16]. ACE2 is expressed on the airway epithelium and underlying vasculature. The SARS-CoV-2 spike protein shares ~76% homology with the spike protein of SARS-CoV-1, the virus responsible for the 2002–2004 SARS outbreak [17]. This is important because the SARS-CoV-1 spike protein requires binding to both ACE2 and cell surface vimentin to enter cells [18], suggesting that SARS-CoV-2 may also interact with cell surface vimentin to bind and infect cells. Therefore, developing a drug that can mimic vimentin and bind to the spike protein may decrease SARS-CoV-2 adhesion to host cells, thus reducing infection.

We previously published on the ability of recombinant vimentin and, more specifically, the rod domain of vimentin (rhRod), to bind P-selectin and block leukocyte adhesion and inflammation in experimental models of sepsis [19,20]. In this study, we hypothesized that rhRod binds to the SARS-CoV-2 spike protein to block viral adhesion and replication. Furthermore, based on our previous work, we hypothesized that treating SARS-CoV-2-infected mice with rhRod will decrease lung inflammation. We aimed to test these hypotheses in this study.

## 2. Results

### 2.1. In Silico Modeling Predicts rhRod Binding to SARS-CoV-2 Spike Protein

**Figure 1:**
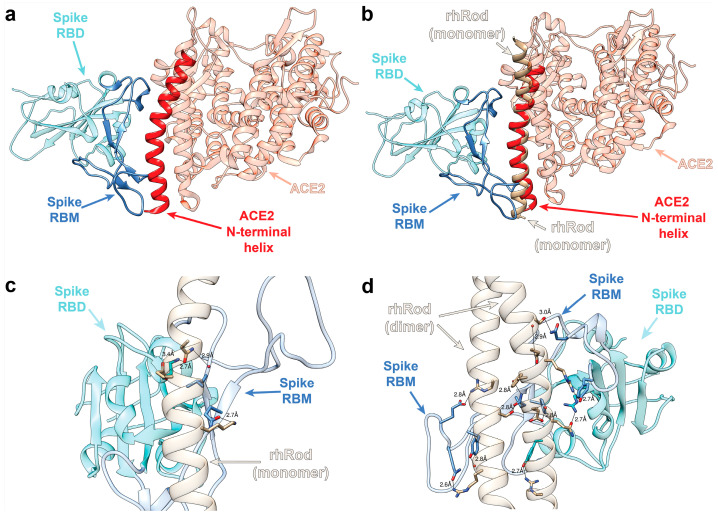
In silico prediction modeling of spike protein RBD, ACE2, and rhRod proteins. (**a**) Structure of spike protein RBD (cyan) in complex with its receptor ACE2 (salmon) (PDB: 6LZG). The spike protein RBM (blue) and *N*-terminal helix of ACE2 (red) are highlighted. (**b**) Overlay of rhRod (tan) between spike protein RBD (cyan) and ACE2 (salmon) proteins based on structural homology between ACE2 and rhRod (PDB:1GK7). The spike protein RBM (blue) and ACE2 *N*-terminal helix (red) are highlighted. (**c**) Representative model of the top-scoring predictions of rhRod monomer and spike protein RBD. (**d**) Representative model of the top scoring predictions of rhRod dimer and spike protein. Orientation of the predicted model in (**d**) closely matched the overlay of rhRod, as seen in (**b**).

Based on previously published data suggesting that the SARS-CoV-1 spike protein binds to host vimentin to enter cells [18], we performed in silico modeling to determine whether the rod domain of vimentin would also bind to the spike protein. Figure 1a shows the relationship between the spike protein receptor binding domain (RBD) and its receptor, ACE2 [15]. Spike protein, or more specifically, an extended loop of the RBD called the receptor-binding motif (RBM), binds to ACE2 via the latter’s *N*-terminal helix. Figure 1b shows an overlay of rhRod at the location of the known ACE2–spike protein interaction, based on the structural homology between rhRod (PDB: 1GK7) and the ACE2 (PDB: 6LZG) *N*-terminal helix. Given the structural homology between rhRod, an alpha-helix that exists in both monomeric and dimeric (coiled-coil) forms, and ACE2 at the interface of the spike protein RBD, we hypothesized that rhRod blocks the binding of spike protein RBM to ACE2 by mimicking the *N*-terminal helix of ACE2. To test this hypothesis, two different protein structures representing a monomer (PDB: 1GK7) and a dimer (PDB: 3TRT) of rhRod were used to simulate the possible protein–protein interactions between rhRod and the spike protein RBD [21]. Our models predict that spike protein–rod interactions are stabilized with three hydrogen bonds and a single salt bridge when the rod domain is a monomer (Figure 1c), whereas there are numerous hydrogen bonds and electrostatic interactions when the rod domain is dimerized (Figure 1d). These results suggest a strong binding capacity of the spike protein RBD to rhRod, particularly the coiled-coil homodimer.

### 2.2. rhRod Binds to SARS-CoV-2 Spike Protein

**Figure 2:**
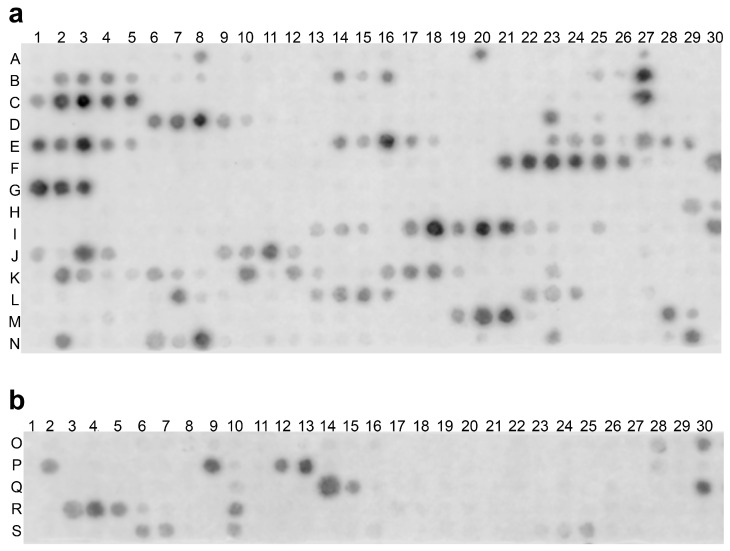
SPOT peptide array of (**a**) SARS-CoV-2 spike protein RBD or (**b**) human vimentin protein, with each spot consisting of 20 aas and adjacent spots frame-shifted by 3 aas. Detection was either with (**a**) IR800-rhRod or (**b**) IR800-S1S2ECD-His. The letters and numbers correspond to the rows and columns, respectively, found in Supplementary Table S1.

Based on our in silico data and published reports suggesting that the SARS-CoV-1 spike protein binds to host vimentin to enter cells [18], we synthesized peptide arrays by SPOT synthesis to determine which amino acid residues play a role in the spike protein and full-length vimentin interaction. Each spot on the array contained 20 amino acid residues from the full-length spike protein (Figure 2a) or vimentin, including the head, rod, and tail domains (Figure 2b); each spot, moving horizontally along a row, was offset by three amino acids from the previous spot, resulting in adjacent spots sharing 17 amino acid residues. The membrane was then probed with either rhRod (Figure 2a) or the spike protein RBD (Figure 2b) to determine to which spots, and thus which amino acids, rhRod or the spike protein bound.

As seen in Figure 2a, we found multiple strings of contiguous spots within the full-length spike protein sequence detected by IR800-rhRod (spot amino acid sequences are shown in Supplementary Table S1), including several clusters in the RBD of the spike protein. These areas, however, did not correlate to the predicted binding regions in our simulation, nor were many residues in the RBM of the spike protein detected using IR800-rhRod. We also found multiple areas within the vimentin protein sequence detected by the extracellular domain of the spike protein RBD (IR800-S1S2ECD-His; Figure 2b). The sequences of vimentin with the best binding to the spike protein RBD, as evidenced by spots with strong detection signals and spatially clustered on the membrane, were all located within the rod domain of vimentin. Many of the residues that were predicted to form hydrogen bonds and electrostatic interactions with the spike protein RBD in the monomer (Figure 1c) and dimer (Figure 1d) were strongly positive and spatially clustered, supporting the proposed model for rhRod.

**Figure 3:**
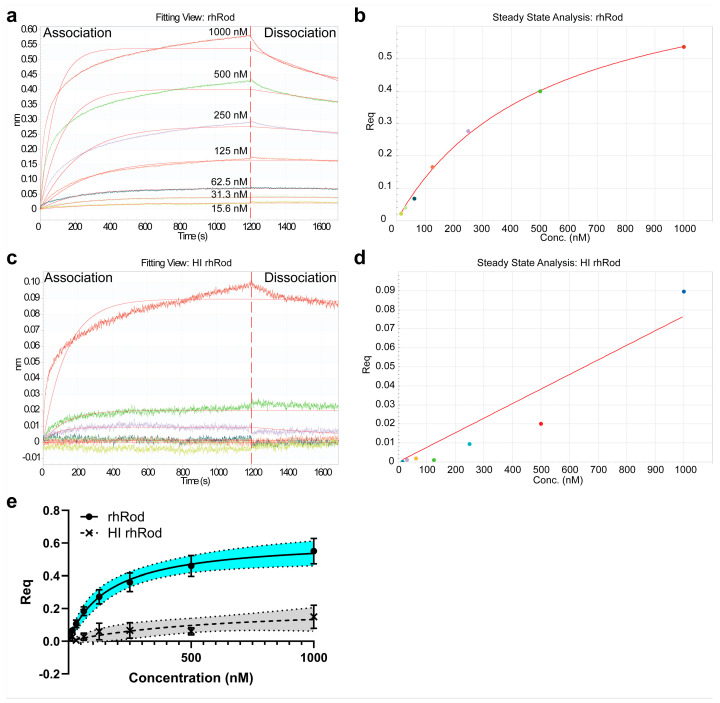
Representative BLI fitting view and steady state analyses of (**a**,**b**) rhRod and (**c**,**d**) HI rhRod. Note the significantly smaller scale for the ordinates for HI rhRod (0–0.1 nm; (**c**,**d**)) compared to rhRod (0–0.6 nm (**a**,**b**)). The colors represent the different rhRod and HI rhRod concentrations (as listed in panel (**a**)). (**e**) Combined non-linear regression curve based on the lot-specific Req at each concentration. Error bars represent SEM and shaded areas indicate the 95% CI of the best-fit line.

Next, we performed biolayer interferometry (BLI) to assess the K_D_ between the recombinant rod domain of vimentin (rhRod) and the SARS-CoV-2 spike protein (Figure 3). We found that rhRod bound to immobilized the SARS-CoV-2 spike protein with great affinity (Table 1), with a combined K_D_ of 163 nM [87, 311] (R^2^ 0.6595; *n* = 7 lots; Figure 3e). Furthermore, to assess whether this interaction required the intact tertiary structure, we heat inactivated rhRod (HI rhRod) and found that HI rhRod either did not bind or bound with a much lower affinity to the SARS-CoV-2 spike protein (622 nM [−3.4 × 10^13^, infinity] (R^2^ 0.2904; *n* = 3 lots; Figure 3a and Table 1) compared to the intact rhRod. This suggests that the intact structure of rhRod is necessary for strong binding to the spike protein.

### 2.3. rhRod Blocks’ SARS-CoV-2 Spike Protein–ACE2 Interactions

**Figure 4:**
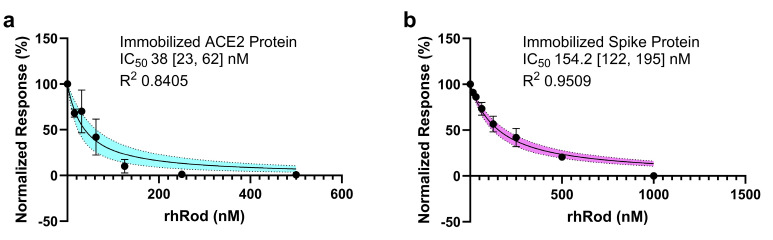
Non-linear regression analyses of the normalized response using BLI to calculate the IC_50_ of rhRod to block spike (S1S2ECD-His)–ACE2 protein interaction. (**a**) ACE2 was immobilized on AR2G sensors and then placed into wells containing 125 µM S1S2ECD-His with increasing concentrations of rhRod (0–500 nM). (**b**) S1S2ECD-His was immobilized on AR2G sensors and first placed into wells containing increasing concentrations of rhRod (0–1000 nM) before wash and placement into wells containing ACE2 (200 nM each). Experiments were performed in duplicate and data are normalized to the highest and lowest response values. Error bars represent SEM and shaded areas represent 95% CI of the best-fit line.

The current understanding of SARS-CoV-2 infection is that the virus mainly enters host cells through the spike protein interacting with the host’s ACE2 [15]. Being able to disrupt this interaction may reduce viral adhesion and entry into the host cells. To test whether rhRod blocked the SARS-CoV-2 spike protein binding to ACE2, we performed binding kinetics assays using two separate methods. Regardless of whether ACE2 (Figure 4a) or the spike (Figure 4b) protein was immobilized, the presence of rhRod decreased the spike protein–ACE2 interactions in a dose-dependent manner.

### 2.4. Daily rhRod Decreases SARS-CoV-2 Replication in Vero E6 Cells

**Figure 5:**
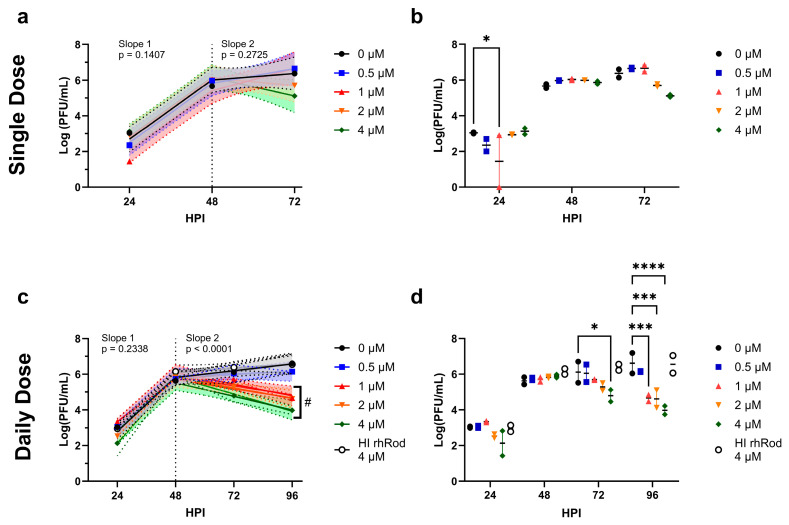
In vitro viral infection studies. Vero E6 cells were cultured in multi-well plates and infected with SARS-CoV-2 (MOI 0.1). (**a**,**b**) Cells treated with a single dose of rhRod at increasing concentrations did not have significant reductions in viral titers at 72 h; however, there was a decrease in plaques after 48 h. (**c**) Cells treated daily with rhRod started having decreased plaque counts after 48 h at rhRod doses of 1 µM and higher. (**d**) At 96 h, daily treatment with rhRod of 1 µM or greater lead to significant decreases in viral titers. Heat inactivated rhRod at the highest dose (4 µM) was similar to no rhRod. # indicates the slopes that were significantly different than the other doses. These data represent analysis in aggregate of two independent infection studies. * *p* < 0.05, *** *p* < 0.001, and **** *p* < 0.0001 using Dunnet’s multiple comparison test versus 0 µM.

Based on our protein-binding kinetic assays and the ability of rhRod to block the spike–ACE2 interactions, we then tested whether rhRod would block SARS-CoV-2 viral replication in vitro. We first tested a single rhRod dose at the time of infection and saw a mild but statistically non-significant decrease in PFU after 48 h (Figure 5a,b). We then performed daily rhRod administration and found that after 48 h, doses of 1 µM and higher were able to decrease viral replication by at least one log (Figure 5c,d).

### 2.5. rhRod Treatment Decreases Lung Inflammation in SARS-CoV-2-Infected K18-hACE2 Mice

**Figure 6:**
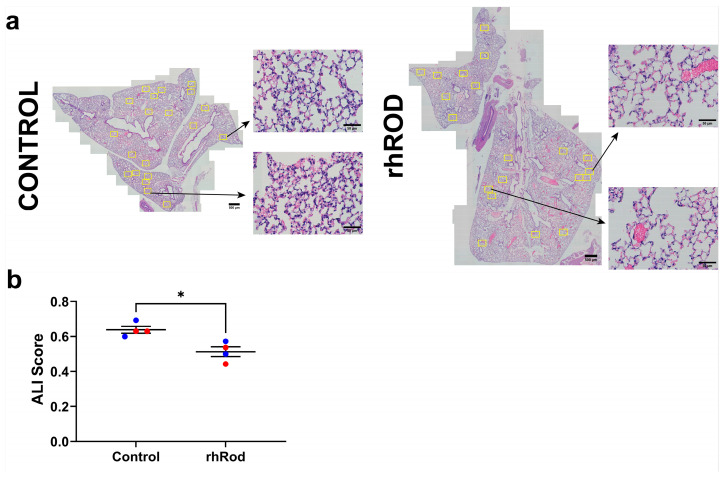
(**a**) Representative lung histology from control- (**left**) and rhRod (**right**)-treated SARS-CoV-2-infected K18-hACE2 mice. Randomly selected sections (yellow boxes) of the lung were used to analyze the degree of acute lung injury at 4 days post-infection. Whole lung section scale bar = 500 µm; Fields of view scale bar = 50 µm. (**b**) Mice treated with rhRod had significantly less lung inflammation than control animals. Red = female and blue = male; * *p* < 0.05.

We previously published on the ability of intraperitoneal recombinant human vimentin to decrease lung inflammation [20] and rhRod to decrease neutrophil recruitment in the liver of endotoxemic mice [19]. Based on those data, we wanted to measure the ability of daily rhRod to decrease lung inflammation in SARS-CoV-2-infected K18-hACE2 mice. As seen in Figure 6, mice receiving rhRod had decreased evidence of lung inflammation at 4 days post-infection compared to those receiving the buffer control (Figure 6). There were no differences in the body weight or viral titers from homogenized lung tissue (Supplementary Figure S1).

## 3. Discussion

These data support that rhRod binds to the extracellular domain of the spike protein and blocks the spike–ACE2 interactions via competitive inhibition. Furthermore, the daily administration of rhRod decreased viral replication after 48 h in vitro. Finally, similar to our previous observation in endotoxemic mice [20], rhRod decreases acute lung injury in SARS-CoV-2-infected animals. Taken together, these findings suggest that rhRod may be useful in attenuating infection and inflammation in patients infected with COVID-19.

To our knowledge, this is the first study to test the rod domain of recombinant vimentin as a potential therapy against SARS-CoV-2 infections in vitro and in vivo. Although the role of vimentin–spike protein interactions have been studied, they have primarily focused on how cell-surface vimentin acts as either a direct receptor or a co-receptor for the spike protein in aiding viral attachment to both endothelial [22] and epithelial cells [23]. Additionally, a recent report used atomic force microscopy to show the strength of the interaction between the SARS-CoV-2 S1 RBD and vimentin, targeting the role of cell-surface vimentin in viral attachment to cells [24]. In these studies, cell-surface vimentin seems to bind to the spike protein with great affinity to allow for viral attachment and cell entry. Their data corroborate our own observations with biolayer interferometry that shows a strong affinity (~163 nM) of spike protein to rhRod. Interestingly, our in vitro data with live, unattenuated ancestral SARS-CoV-2 (USA-WA1/2020) support that rhRod, when given daily, decreases viral replication after 48 h in Vero E6 epithelial cells. This may be due to various factors, including the hypothesis that rhRod blocks the interaction between spike and ACE2 by exhibiting structural similarity to the latter ligand. Another mechanism for viral inhibition may be that rhRod acts as a decoy ligand for the SARS-CoV-2 spike protein, preventing it from binding to cell-surface vimentin. We expect the latter to be less likely, as the proposed interaction between the spike protein and cell-surface vimentin occurs at the C-terminal domain of full-length vimentin, which did not display binding in our SPOT data and is absent from rhRod [24]. Finally, rhRod may be binding to cell-surface vimentin itself, since vimentin naturally forms oligomers [25], to prevent the spike protein from interacting. Further studies are needed to determine which of these are the primary action through which rhRod decreases viral replication in vitro.

In addition to our findings in vitro, we observed that rhRod decreased leukocytic infiltration into the lungs of SARS-CoV-2-infected mice. These data are similar to our observations that recombinant vimentin and rhRod decreases leukocytic infiltration in the lungs [20] and liver [19], respectively, in endotoxemic mice. We had previously reported that both recombinant vimentin and its rod domain bind to P-selectin with high affinity to block P-selectin–glycoprotein ligand-1 interactions. This leads to a decrease in leukocyte rolling and firm adhesion to an inflamed endothelium under flowing conditions. We presume that the beneficial effect of rhRod in SARS-CoV-2-infected mice is similar due to the virus’ ability to activate platelets [26] and the endothelium [27] to increase P-selectin expression, which leads to neutrophil activation and capture. rhRod may then decrease neutrophil–platelet–endothelial interactions to attenuate lung injury. Although lung viral titers were similar in both groups, we and others have published that SARS-CoV-2 viral loads are not associated with the severity of clinical illness or lung computed tomography findings of illness severity [28,29,30,31]. Additionally, our model was designed to identify lung inflammation at 4 days, similar to our previous methods with endotoxin-induced acute lung injury [20,32]. To understand how this reduced inflammation translates to the remaining body systems and disease outcomes, future studies will be necessary to evaluate whether rhRod can attenuate inflammation-induced injury in other organ systems.

While these data provide evidence to support the further exploration of rhRod as a treatment for SARS-CoV-2 infections, our study had limitations. There are limitations in the peptide array data, particularly for the spike protein RBD. The RBM of the spike protein RBD is an extended loop that creates a large pocket for contact with ACE2. Unfortunately, peptide arrays are limited in the secondary and tertiary structures that can be formed. Many of the spike protein residues that were predicted to bind to rhRod by in silico modeling are in close physical proximity, but distal through the primary amino acid sequence. As such, there are no spots that contain more than a single residue that were predicted to bind to rhRod, as these residues were all more than 20 amino acids apart. This may explain why our in silico model was able to accurately predict the rhRod residues that bind to the spike protein RBD by the peptide array, but not the reverse, as rhRod is a helical structure where amino acids residues that are spatially close are sequentially close by primary sequence. Additionally, although the use of VeroE6 cells has been a validated and well-documented in vitro model for studying the SARS-CoV-2 life cycle and performing early drug screening studies, previous studies highlight that not all VeroE6 results are translatable to human use [33,34,35]. Therefore, validating the results against cell lines from other species will be valuable in future studies. Our in silico, SPOT peptide array, and biolayer interferometry data suggest that rhRod blocks spike protein–ACE2 interactions; therefore, VeroE6 cells were the most reasonable model for the initial testing of rhRod. Finally, our in vivo studies focused on lung inflammation, which typically occurs before death. Future studies will be needed to test whether rhRod affects mortality in SARS-CoV-2-infected animals.

These studies suggest that rhRod decreases SARS-CoV-2 replication in vitro and lung inflammation in vivo. Future studies will need to evaluate the protective effects of rhRod against additional viral variants, identify the optimal dosing scheme that prevents both viral replication and host lung injury, and evaluate the effect on the survival and long-term sequelae in small animal models.

## 4. Materials and Methods

### 4.1. Study Approval

This study was approved by the R&D Committee at the Michael E. DeBakey Veterans Affairs Medical Center. All animal research was conducted at Baylor College of Medicine (BCM) and approved by the BCM Institutional Animal Use and Care Committee (AN-8492).

### 4.2. In Silico Modeling of rhRod–Spike Protein Interactions

The interaction between rhRod and SARS-CoV-2 spike glycoprotein receptor binding domain (RBD) was simulated using the SwarmDock interface prediction server using full blind docking prediction [21]. The receptor chosen was chain B, representing the spike protein RBD, from a spike RBD–ACE2 structure (PDB:6LZG), and two structures of the rod domain of vimentin (PDB:1GK7 and 3TRT) were chosen as ligands to represent rhRod. Also, 3TRT is a homodimer while 1GK7 is a monomer. Each ligand’s binding to the RBD of spike was simulated, and the top 10 structures were compared. A representative structure that best represented the majority of the predicted structures for each ligand was chosen for graphical viewing. The models are shown using Chimera software (1.16, UCSF).

### 4.3. Creation of Recombinant Rod Domain of Vimentin (rhRod)

The rod domain of vimentin was synthesized using sequence NM_003380.3 (National Center for Biotechnology Information) from base pairs 717–1652 (corresponding to amino acid residues 96–407). The sequence was inserted into pQE-30 vector (GenScript, Piscataway, NJ, USA) and the resultant plasmid was transformed into *Escherichia coli* (Strain M15). *E*. *coli* harboring rhRod pQE-30 was grown to mid-log phase at 37 °C and protein expression was induced using 1.5 mM isopropyl-β-D-1-thiogalactopyranoside (IPTG) for 4 h. The *E*. *coli* was pelleted and lysed to obtain inclusion bodies which were then solubilized with 6 M guanidine hydrochloride, 25 mM Tris, pH 7.4, and supplemented with protease inhibitor at 4 °C overnight. Cell lysates were centrifuged at 19,000 rpm for 30 min and the supernatants filtered through a 0.45 µm filter prior to loading onto Ni^2+^ affinity columns previously equilibrated with binding buffer (7 M urea, 50 mM Tris, pH 8). Bound proteins were then eluted with elution buffer (500 mM imidazole, 7 M urea, 50 mM Tris, pH 8). The purified sample was serially dialyzed against decreasing concentrations of urea in Tris buffer at 4 °C. Samples were ultimately dialyzed twice against 20 mM sodium phosphate buffer, pH 8, and then filtered through a 0.2 µm filter under sterile conditions. The protein concentration was determined by measuring absorbance at 280 nm, using an extinction coefficient of 15,350 L/mol/cm and molecular weight of 38.2 kDa. Purity was confirmed using SDS-PAGE and Coomassie blue stain as well as Western blot. In some experiments, aliquots of rhRod were denatured through heat inactivation by boiling in water for 10 min (100 °C), as we did before [19].

### 4.4. Biolayer Interferometry

Protein–protein affinity was determined using biolayer interferometry on the Octet Red^384^ (Sartorius, Ann Arbor, MI, USA), like we did before [19,20]. To measure binding kinetics, we immobilized recombinant spike S1S2 extracellular domain of SARS-CoV-2 (S1S2ECD-His; 10 µg/mL in 10 mM sodium acetate, pH 4; SinoBiological US, Houston, TX, USA) on amine reactive 2nd-generation sensors (AR2G; Sartorius, Ann Arbor, MI, USA) using 10 mM sulfo-*N*-hydroxysuccinimide and 20 mM 1-ethyl-3-(3-dimethylaminopropyl)carbodiimide (Thermo Scientific, Waltham, MA, USA). The amine-coupling reaction was quenched using 1 M ethanolamine, pH 8.5 (Sartorius, Ann Arbor, MI, USA). Sensors were regenerated with 10 mM glycine, pH 1.75, between assays. Spike-immobilized sensors were then used to assess binding kinetics to both rhRod and heat-inactivated rhRod. Multiple different lots of rhRod, created on different days, were tested to ensure that the effects seen were not lot-dependent. K_D_ (best-fit value and 95% CI) and R^2^ were calculated in GraphPad Prism 10 software.

To measure the IC_50_ of rhRod to block S1S2ECD-His–ACE2 receptor protein interaction, we performed 2 different assays. First, we immobilized ACE2 receptor protein (Sigma-Aldrich, Burlington, MA, USA; 9 µg/mL) onto AR2G sensors, like above. We then determined the K_D_ of spike protein (130 ± 20 nM; R^2^ 0.9797) and rhRod (780 ± 210 nM; R^2^ 0.9792) alone to immobilize ACE2. Next, we combined a standard concentration of spike protein (125 nM) with increasing concentrations of rhRod (0–500 nM). Finally, we plotted the normalized responses against increasing concentrations of rhRod mixed with spike protein. In the second assay, we immobilized S1S2ECD-His onto AR2G sensors, like above. Next, spike-loaded sensors were dipped into wells containing increasing concentrations of rhRod (0–1000 nM). After excess rhRod was washed off, sensors were placed into wells containing 200 nM ACE2 protein. Finally, we plotted the normalized responses. All experiments were performed in duplicate and the IC_50_ (best-fit and 95% CI) was calculated in GraphPad Prism 10 software.

### 4.5. SPOT Peptide Array

To determine potential interaction sites between recombinant vimentin and spike S1S2-ECD, we synthesized SPOT peptide arrays on derivatized cellulose membranes using the MultiPep RS-automated peptide synthesizer (CEM, Matthews, NC, USA), as previously described [19,36]. We used the full-length sequence of vimentin (NCBI reference sequence NP_003371.1) and spike protein (NCBI reference sequence YP_009724390.1) for each array. Peptide spots of both proteins were 20 aas long and adjacent spots were frame-shifted by 3 aas (Supplementary Table S1). Detection probes were created by labeling S1S2ECD-His or rhRod with IRDye 800CW NHS Ester (LiCor Biosciences, Lincoln, NE, USA), per manufacturer’s recommendation, followed by dialysis against Dulbecco’s phosphate-buffered saline to remove free dye. Once peptide arrays were completed, membranes were rewetted in 100% methanol for 10 min, after which they were washed with wash buffer (Tris-buffered saline, 0.05% (*v*/*v*) Tween-20, 0.1% bovine serum albumin) three times. Washed membranes were placed in LiCor Odyssey Intercept Blocking Buffer (TBS; LiCor Biosciences) for 4 h at room temperature. Blocked membranes were then washed three times prior to incubation with labeled detection probes (1 µg/mL) for 1 h at room temperature. Membranes were then washed three times prior to imaging on a Li-Cor Odyssey (LiCor Biosciences, Lincoln, NE, USA) using the 800 nm channel for 5 min.

### 4.6. Viral and Cell Culture Stocks

Vero E6 cells (*Cercopithecus aethiops*, Vero 76, clone E6, ATCC CRL-1586, Manassas VA, USA) were maintained in complete DMEM supplemented with 10%FBS and 1% pen/step. SARS-CoV-2 (USA-WA1/2020) was obtained from the University of Texas Medical Branch World Reference Center for Emerging Viruses and Arboviruses and passaged once in Vero E6 cells. This stock was titered by standard plaque assay on Vero E6 cells as previously described [37]. The virus stock was sequenced prior to use in the studies to verify its identity with the parent strain. All work involving live SARS-CoV-2 was performed under biosafety level 3 (BSL-3) conditions in the Feigin Tower Pathogen Resource Core.

### 4.7. Viral Infection Studies

Vero E6 cells were infected in multi-well plates with a multiplicity of infection (MOI) of 0.1 and exposed to rhRod (0.5, 1, 2, and 4), heat-inactivated rhRod (4 µM), or buffer only at 24 h intervals. At each timepoint, supernatant was removed/collected and replaced with fresh rhRod or control buffer, and plaque assays were completed in triplicate, as previously described [38]. This study was repeated such that two independent replicates were performed on different dates and the results were analyzed in aggregate.

### 4.8. In Vivo Animal Studies

Male and female 6-week old K18-hACE2 mice (B6.Cg-Tg(K18-ACE2)2Prlmm/J; #034860; The Jackson Laboratory, Bar Harbor, ME, USA) were housed in individually ventilated cages under BSL-3 conditions [37,39,40]. Mice (5 females and 5 males per group) were infected intranasal with 1 × 10^4^ pfu of SARS-CoV-2. Mice were monitored daily for weight loss and other signs of disease over 4 days post-infection, at which time lung tissue was collected for viral titers. Weight change was determined as percent of pre-infection weight (day −1). At necropsy, one lung from each animal was collected for viral titration. Briefly, lungs were homogenized and a plaque assay was performed as previously described [38]. In a subset of animals (the first 2 females and 2 males per group), the other lung was inflated, removed, and formalin-fixed for removal from BSL-3. Formalin-fixed paraffin-embedded lungs were sectioned and stained with hematoxylin and eosin. A blinded investigator scored the H&E sections for histologic acute lung injury based on the methods defined by the American Thoracic Society [41]. Whole lung sections were scanned using an Olympus FV 3000 with a 20X objective (NA 0.7). The sections were traced in Fiji/ImageJ 1.54 h and regions of interest (640 × 480 pixels) were randomly selected within the traced outline using a macro for analysis (Supplemental Methods). Twenty consecutive fields of view (FOV) were used to calculate the lung injury score, with FOV containing <50% alveoli excluded, as we have done before [20].

### 4.9. Statistical Analysis

All statistics were performed in GraphPad Prism 10 (San Diego, CA, USA). K_D,_ IC_50,_ and plaque assays’ determination was performed using non-linear regression analysis with least squared regression, as stated above. Non-linear regression, K_D_, and IC_50_ are reported as the best-fit value with asymmetrical 95% confidence intervals. Viral titers that were 0 were changed to 1 (1 animal per group) prior to log_10_-transformation and analysis via Student’s *t*-test. Statistical analyses of acute lung injury scores were performed using Student’s *t*-test and represented as mean ± SEM.

## 5. Patents

FWL in an inventor on a patent for recombinant vimentin (US Patent # 10,695,401).

## Figures and Tables

**Table 1:** K_D_ of individual lots of rhRod to immobilized spike protein (S1S2ECD-His).

Lot	K_D_ [95% CI] (nM)	R^2^
rhRod-1	156 [115, 212]	0.9722
rhRod-2	384 [168, 1000]	0.9584
rhRod-3	191 [98, 381]	0.8976
rhRod-4	175 [90, 350]	0.7787
rhRod-5	135 [97, 188]	0.9662
rhRod-6	227 [126, 425]	0.977
rhRod-7	104 [57, 188]	0.9636
*HI rhRod-2	Indeterminate	0.8819
*HI-rhRod-3	146 [−2657, infinity]	0.3646
*HI rhRod-4	525 [181, 2439]	0.9412

*HI denotes heat-inactivated version of the stated rhRod lot. Each run had 8 concentrations and each lot had 1–4 technical replicates.

## Data Availability

Data care contained within the article and Supplementary Materials, as well as upon request.

## Supplementary Materials

The supporting information can be downloaded at: https://www.mdpi.com/article/10.3390/ijms25052477/s1.
