# Supplementary material for: Recombinant Rod Domain of Vimentin Reduces SARS-CoV-2 Viral Replication by Blocking Spike Protein–ACE2 Interactions
Source: Int J Mol Sci. 2024 Feb 20;25(5):2477. doi: 10.3390/ijms25052477 (PMC10931652; doi:10.3390/ijms25052477)
Supplement: Supplementary file 1 [file ijms-25-02477-s001.zip › Supplemental Figure.pdf]

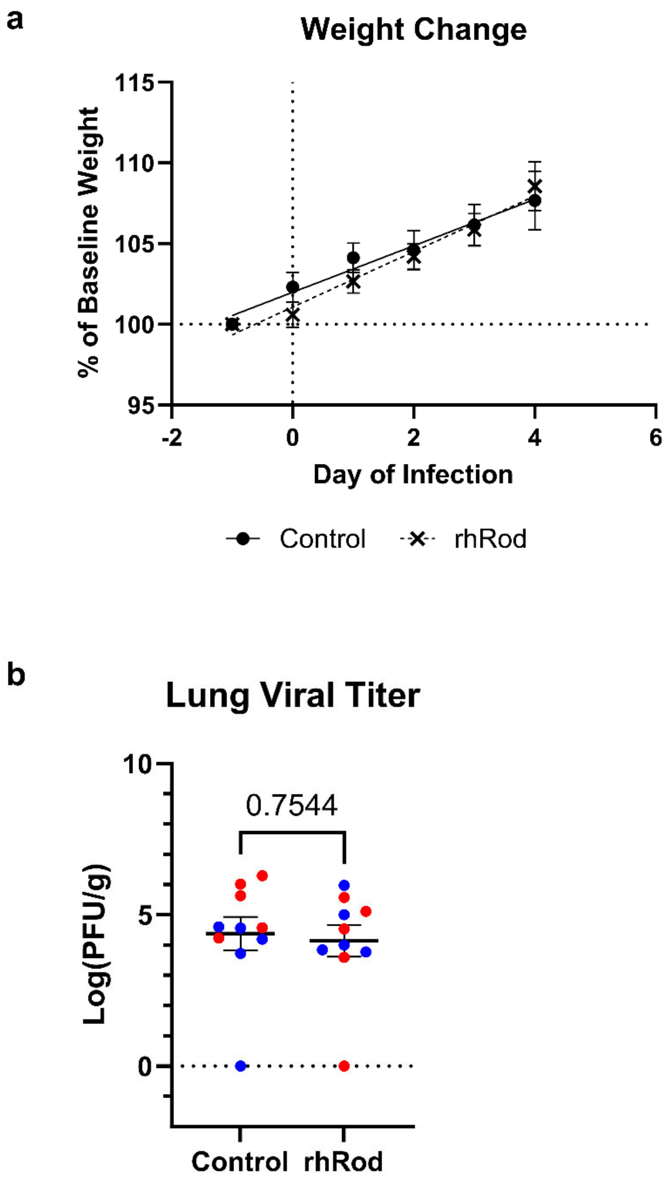

Supplemental Figure S1. (a) Weight change of SARS-CoV-2-infected mice was similar between groups. (b) Log_{10}-transformed viral titers from homogenized lung tissue was similar between groups. Red and blue circles represent female and male animals, respectively.
