# Supplementary material for: Recombinant Rod Domain of Vimentin Reduces SARS-CoV-2 Viral Replication by Blocking Spike Protein–ACE2 Interactions
Source: Int J Mol Sci. 2024 Feb 20;25(5):2477. doi: 10.3390/ijms25052477 (PMC10931652; doi:10.3390/ijms25052477)

Supplemental Table 1: Spot Membrane Results
Bolded Regions Correspond to ≥3 contiguous spots detected

SPOT: Spike Protein			DETECT: IR800/rhRod
1	A 1	2224.8	MFVFLVLLPLVSSQCVNLTT
2	A 2	2232.7	FLVLLPLVSSQCVNLTTRTQ
3	A 3	2180.6	LLPLVSSQCVNLTTRTQLPP
4	A 4	2192.5	LVSSQCVNLTTRTQLPPAYT
5	A 5	2241.5	SQCVNLTTRTQLPPAYTNSF
6	A 6	2237.6	VNLTTRTQLPPAYTNSFTRG
7	A 7	2336.7	TTRTQLPPAYTNSFTRGVYY
8	A 8	2318.7	TQLPPAYTNSFTRGVYYPDK
9	A 9	2378.8	PPAYTNSFTRGVYYPDKVFR
10	A10	2386.8	YTNSFTRGVYYPDKVFRSSV
11	A11	2345.8	SFTRGVYYPDKVFRSSVLHS
12	A12	2354.7	RGVYYPDKVFRSSVLHSTQD
13	A13	2415.9	YYPDKVFRSSVLHSTQDLFL
14	A14	2383.9	DKVFRSSVLHSTQDLFLPFF
15	A15	2341.8	FRSSVLHSTQDLFLPFFSNV
16	A16	2385.8	SVLHSTQDLFLPFFSNVTWF
17	A17	2407.8	HSTQDLFLPFFSNVTWFHAI
18	A18	2405.8	QDLFLPFFSNVTWFHAIHVS
19	A19	2321.7	FLPFFSNVTWFHAIHVSGTN
20	A20	2250.6	FFSNVTWFHAIHVSGTNGTK
21	A21	2287.6	NVTWFHAIHVSGTNGTKRFD
22	A22	2283.6	WFHAIHVSGTNGTKRFDNPV
23	A23	2170.6	AIHVSGTNGTKRFDNPVLPF
24	A24	2135.5	VSGTNGTKRFDNPVLPFNDG
25	A25	2301.7	TNGTKRFDNPVLPFNDGVYF
26	A26	2288.7	TKRFDNPVLPFNDGVYFAST
27	A27	2247.6	FDNPVLPFNDGVYFASTEKS
28	A28	2211.7	PVLPFNDGVYFASTEKSNII
29	A29	2301.8	PFNDGVYFASTEKSNIIRGW
30	A30	2260.9	DGVYFASTEKSNIIRGWIFG
31	B 1	2305	YFASTEKSNIIRGWIFGTTL
**32**	**B 2**	**2253.9**	**STEKSNIIRGWIFGTTLDSK**
**33**	**B 3**	**2252.9**	**KSNIIRGWIFGTTLDSKTQS**
**34**	**B 4**	**2263.1**	**IIRGWIFGTTLDSKTQSLLI**
**35**	**B 5**	**2207.8**	**GWIFGTTLDSKTQSLLIVNN**
36	B 6	2137.6	FGTTLDSKTQSLLIVNNATN
37	B 7	2143.6	TLDSKTQSLLIVNNATNVVI
38	B 8	2144.6	SKTQSLLIVNNATNVVIKVC
39	B 9	2232.6	QSLLIVNNATNVVIKVCEFQ
40	B10	2268.6	LIVNNATNVVIKVCEFQFCN
41	B11	2302.5	NNATNVVIKVCEFQFCNDPF
42	B12	2272.6	TNVVIKVCEFQFCNDPFLGV
43	B13	2421.8	VIKVCEFQFCNDPFLGVYYH
**44**	**B14**	**2437.7**	**VCEFQFCNDPFLGVYYHKNN**
**45**	**B15**	**2507.9**	**FQFCNDPFLGVYYHKNNKSW**
**46**	**B16**	**2432.8**	**CNDPFLGVYYHKNNKSWMES**
47	B17	2533	PFLGVYYHKNNKSWMESEFR
48	B18	2524.9	GVYYHKNNKSWMESEFRVYS
49	B19	2477.8	YHKNNKSWMESEFRVYSSAN
50	B20	2367.6	NNKSWMESEFRVYSSANNCT
51	B21	2450.7	SWMESEFRVYSSANNCTFEY
52	B22	2360.5	ESEFRVYSSANNCTFEYVSQ
53	B23	2372.7	FRVYSSANNCTFEYVSQPFL
54	B24	2329.7	YSSANNCTFEYVSQPFLMDL
55	B25	2306.7	ANNCTFEYVSQPFLMDLEGK
56	B26	2306.7	CTFEYVSQPFLMDLEGKQGN
57	B27	2344.8	EYVSQPFLMDLEGKQGNFKN
58	B28	2351.9	SQPFLMDLEGKQGNFKNLRE
59	B29	2433.1	FLMDLEGKQGNFKNLREFVF
60	B30	2397	DLEGKQGNFKNLREFVFKNI
**61**	**C 1**	**2375**	**GKQGNFKNLREFVFKNIDGY**
**62**	**C 2**	**2450.2**	**GNFKNLREFVFKNIDGYFKI**
**63**	**C 3**	**2510.3**	**KNLREFVFKNIDGYFKIYSK**
**64**	**C 4**	**2490.1**	**REFVFKNIDGYFKIYSKHTP**
**65**	**C 5**	**2398.1**	**VFKNIDGYFKIYSKHTPINL**
66	C 6	2394	NIDGYFKIYSKHTPINLVRD
67	C 7	2390	GYFKIYSKHTPINLVRDLPQ
68	C 8	2313.9	KIYSKHTPINLVRDLPQGFS
69	C 9	2222.7	SKHTPINLVRDLPQGFSALE
70	C10	2179.7	TPINLVRDLPQGFSALEPLV
71	C11	2193.7	NLVRDLPQGFSALEPLVDLP
72	C12	2150.8	RDLPQGFSALEPLVDLPIGI
73	C13	2094.7	PQGFSALEPLVDLPIGINIT
74	C14	2243.9	FSALEPLVDLPIGINITRFQ
75	C15	2266	LEPLVDLPIGINITRFQTLL
76	C16	2248	LVDLPIGINITRFQTLLALH
77	C17	2327.1	LPIGINITRFQTLLALHRSY
78	C18	2315	GINITRFQTLLALHRSYLTP
79	C19	2289.9	ITRFQTLLALHRSYLTPGDS
80	C20	2150.7	FQTLLALHRSYLTPGDSSSG
81	C21	2132.7	LLALHRSYLTPGDSSSGWTA
82	C22	2034.5	LHRSYLTPGDSSSGWTAGAA
83	C23	2025.5	SYLTPGDSSSGWTAGAAAYY
84	C24	1981.4	TPGDSSSGWTAGAAAYYVGY
85	C25	2064.5	DSSSGWTAGAAAYYVGYLQP
86	C26	2179.7	SGWTAGAAAYYVGYLQPRTF
87	C27	2203.9	TAGAAAYYVGYLQPRTFLLK
88	C28	2381	AAAYYVGYLQPRTFLLKYNE
89	C29	2440	YYVGYLQPRTFLLKYNENGT
90	C30	2343.9	GYLQPRTFLLKYNENGTITD
91	D 1	2295.7	QPRTFLLKYNENGTITDAVD
92	D 2	2201.7	TFLLKYNENGTITDAVDCAL
93	D 3	2165.6	LKYNENGTITDAVDCALDPL
94	D 4	2078.3	NENGTITDAVDCALDPLSET
95	D 5	2053.4	GTITDAVDCALDPLSETKCT
**96**	**D 6**	**2110.5**	**TDAVDCALDPLSETKCTLKS**
**97**	**D 7**	**2170.6**	**VDCALDPLSETKCTLKSFTV**
**98**	**D 8**	**2167.7**	**ALDPLSETKCTLKSFTVEKG**
**99**	**D 9**	**2272.8**	**PLSETKCTLKSFTVEKGIYQ**
**100**	**D10**	**2277.7**	**ETKCTLKSFTVEKGIYQTSN**
101	D11	2321.8	CTLKSFTVEKGIYQTSNFRV
102	D12	2330.7	KSFTVEKGIYQTSNFRVQPT
103	D13	2297.6	TVEKGIYQTSNFRVQPTESI
104	D14	2370.8	KGIYQTSNFRVQPTESIVRF
105	D15	2396.7	YQTSNFRVQPTESIVRFPNI
106	D16	2332.7	SNFRVQPTESIVRFPNITNL
107	D17	2331.7	RVQPTESIVRFPNITNLCPF
108	D18	2233.6	PTESIVRFPNITNLCPFGEV
109	D19	2238.7	SIVRFPNITNLCPFGEVFNA
110	D20	2343.8	RFPNITNLCPFGEVFNATRF
111	D21	2200.6	NITNLCPFGEVFNATRFASV
112	D22	2292.7	NLCPFGEVFNATRFASVYAW
113	D23	2360.8	PFGEVFNATRFASVYAWNRK
114	D24	2415.9	EVFNATRFASVYAWNRKRIS
115	D25	2356.8	NATRFASVYAWNRKRISNCV
116	D26	2419.9	RFASVYAWNRKRISNCVADY
117	D27	2344.8	SVYAWNRKRISNCVADYSVL
118	D28	2359.8	AWNRKRISNCVADYSVLYNS
119	D29	2293.8	RKRISNCVADYSVLYNSASF
120	D30	2188.6	ISNCVADYSVLYNSASFSTF
121	E 1	2268.7	CVADYSVLYNSASFSTFKCY
122	E 2	2238.7	DYSVLYNSASFSTFKCYGVS
123	E 3	2199.7	VLYNSASFSTFKCYGVSPTK
124	E 4	2166.6	NSASFSTFKCYGVSPTKLND
125	E 5	2257.8	SFSTFKCYGVSPTKLNDLCF
126	E 6	2250.7	TFKCYGVSPTKLNDLCFTNV
127	E 7	2223.6	CYGVSPTKLNDLCFTNVYAD
128	E 8	2233.6	VSPTKLNDLCFTNVYADSFV
129	E 9	2276.8	TKLNDLCFTNVYADSFVIRG
130	E10	2277.6	NDLCFTNVYADSFVIRGDEV
131	E11	2332.7	CFTNVYADSFVIRGDEVRQI
132	E12	2206.6	NVYADSFVIRGDEVRQIAPG
133	E13	2116.5	ADSFVIRGDEVRQIAPGQTG
**134**	**E14**	**2155.7**	**FVIRGDEVRQIAPGQTGKIA**
**135**	**E15**	**2188.6**	**RGDEVRQIAPGQTGKIADYN**
**136**	**E16**	**2264.8**	**EVRQIAPGQTGKIADYNYKL**
**137**	**E17**	**2207.7**	**QIAPGQTGKIADYNYKLPDD**
**138**	**E18**	**2200.7**	**PGQTGKIADYNYKLPDDFTG**
139	E19	2233.8	TGKIADYNYKLPDDFTGCVI
140	E20	2318.8	IADYNYKLPDDFTGCVIAWN
141	E21	2334.7	YNYKLPDDFTGCVIAWNSNN
142	E22	2209.6	KLPDDFTGCVIAWNSNNLDS
**143**	**E23**	**2155.5**	**DDFTGCVIAWNSNNLDSKVG**
**144**	**E24**	**2112.5**	**TGCVIAWNSNNLDSKVGGNY**
**145**	**E25**	**2241.7**	**VIAWNSNNLDSKVGGNYNYL**
146	E26	2390.9	WNSNNLDSKVGGNYNYLYRL
**147**	**E27**	**2435.1**	**NNLDSKVGGNYNYLYRLFRK**
**148**	**E28**	**2408.1**	**DSKVGGNYNYLYRLFRKSNL**
**149**	**E29**	**2450.2**	**VGGNYNYLYRLFRKSNLKPF**
150	E30	2637.3	NYNYLYRLFRKSNLKPFERD
151	F 1	2547.3	YLYRLFRKSNLKPFERDIST
152	F 2	2513.2	RLFRKSNLKPFERDISTEIY
153	F 3	2352.9	RKSNLKPFERDISTEIYQAG
154	F 4	2266.7	NLKPFERDISTEIYQAGSTP
155	F 5	2185.5	PFERDISTEIYQAGSTPCNG
156	F 6	2097.4	RDISTEIYQAGSTPCNGVEG
157	F 7	2077.3	STEIYQAGSTPCNGVEGFNC
158	F 8	2167.5	IYQAGSTPCNGVEGFNCYFP
159	F 9	2091.4	AGSTPCNGVEGFNCYFPLQS
160	F10	2243.6	TPCNGVEGFNCYFPLQSYGF
161	F11	2268.6	NGVEGFNCYFPLQSYGFQPT
162	F12	2268.6	EGFNCYFPLQSYGFQPTNGV
163	F13	2283.6	NCYFPLQSYGFQPTNGVGYQ
164	F14	2319.7	FPLQSYGFQPTNGVGYQPYR
165	F15	2259.5	QSYGFQPTNGVGYQPYRVVV
166	F16	2228.6	GFQPTNGVGYQPYRVVVLSF
167	F17	2251.7	PTNGVGYQPYRVVVLSFELL
168	F18	2244.7	GVGYQPYRVVVLSFELLHAP
169	F19	2302.7	YQPYRVVVLSFELLHAPATV
170	F20	2171.6	YRVVVLSFELLHAPATVCGP
**171**	**F21**	**2096.6**	**VVLSFELLHAPATVCGPKKS**
**172**	**F22**	**2113.6**	**SFELLHAPATVCGPKKSTNL**
**173**	**F23**	**2091.6**	**LLHAPATVCGPKKSTNLVKN**
**174**	**F24**	**2058.5**	**APATVCGPKKSTNLVKNKCV**
**175**	**F25**	**2194.6**	**TVCGPKKSTNLVKNKCVNFN**

**176**	**F26**	**2209.7**	**GPKKSTNLVKNKCVNFNFNG**
177	F27	2198.7	KSTNLVKNKCVNFNFNGLTG
178	F28	2139.6	NLVKNKCVNFNFNGLTGTGV
179	F29	2156.6	KNKCVNFNFNGLTGTGVLTE
**180**	**F30**	**2115.5**	**CVNFNFNGLTGTGVLTESNK**
**181**	**G 1**	**2187.8**	**FNFNGLTGTGVLTESNKKFL**
**182**	**G 2**	**2151.7**	**NGLTGTGVLTESNKKFLPFQ**
**183**	**G 3**	**2199.7**	**TGTGVLTESNKKFLPFQQFG**
184	G 4	2324.9	GVLTESNKKFLPFQQFGRDI
185	G 5	2342.8	TESNKKFLPFQQFGRDIADT
186	G 6	2312.8	NKKFLPFQQFGRDIADTTDA
187	G 7	2312.7	FLPFQQFGRDIADTTDAVRD
188	G 8	2281.5	FQQFGRDIADTTDAVRDPQT
189	G 9	2233.6	FGRDIADTTDAVRDPQTLEI
190	G10	2214.6	DIADTTDAVRDPQTLEILDI
191	G11	2216.5	DTTDAVRDPQTLEILDITPC
192	G12	2190.6	DAVRDPQTLEILDITPCSFG
193	G13	2148.6	RDPQTLEILDITPCSFGGVS
194	G14	2093.6	QTLEILDITPCSFGGVSVIT
195	G15	2006.5	EILDITPCSFGGVSVITPGT
196	G16	1953.3	DITPCSFGGVSVITPGTNTS
197	G17	1965.2	PCSFGGVSVITPGTNTSNQV
198	G18	1961.3	FGGVSVITPGTNTSNQVAVL
199	G19	2106.3	VSVITPGTNTSNQVAVLYQD
200	G20	2137.3	ITPGTNTSNQVAVLYQDVNC
201	G21	2155.2	GTNTSNQVAVLYQDVNCTEV
202	G22	2150.2	TSNQVAVLYQDVNCTEVPVA
203	G23	2169.3	QVAVLYQDVNCTEVPVAIHA
204	G24	2227.4	VLYQDVNCTEVPVAIHADQL
205	G25	2151.2	QDVNCTEVPVAIHADQLTPT
206	G26	2250.4	NCTEVPVAIHADQLTPTWRV
207	G27	2283.5	EVPVAIHADQLTPTWRVYST
208	G28	2216.5	VAIHADQLTPTWRVYSTGSN
209	G29	2307.5	HADQLTPTWRVYSTGSNVFQ
210	G30	2312.6	QLTPTWRVYSTGSNVFQTRA
211	H 1	2243.6	PTWRVYSTGSNVFQTRAGCL
212	H 2	2100.6	RVYSTGSNVFQTRAGCLIGA
213	H 3	2047.4	STGSNVFQTRAGCLIGAEHV
214	H 4	2117.4	SNVFQTRAGCLIGAEHVNNS
215	H 5	2212.5	FQTRAGCLIGAEHVNNSYEC
216	H 6	2161.5	RAGCLIGAEHVNNSYECDIP
217	H 7	2118.5	CLIGAEHVNNSYECDIPIGA
218	H 8	2062.4	GAEHVNNSYECDIPIGAGIC
219	H 9	2126.5	HVNNSYECDIPIGAGICASY
220	H10	2133.5	NSYECDIPIGAGICASYQTQ
221	H11	2071.4	ECDIPIGAGICASYQTQTNS
222	H12	2133.6	IPIGAGICASYQTQTNSPRR
223	H13	2124.5	GAGICASYQTQTNSPRRARS
224	H14	2196.5	ICASYQTQTNSPRRARSVAS
225	H15	2237.5	SYQTQTNSPRRARSVASQSI
226	H16	2206.6	TQTNSPRRARSVASQSIIAY
227	H17	2195.7	NSPRRARSVASQSIIAYTMS
228	H18	2138.8	RRARSVASQSIIAYTMSLGA
229	H19	2085.6	RSVASQSIIAYTMSLGAENS
230	H20	2076.6	ASQSIIAYTMSLGAENSVAY
231	H21	2105.6	SIIAYTMSLGAENSVAYSNN
232	H22	2063.5	AYTMSLGAENSVAYSNNSIA
233	H23	2039.5	MSLGAENSVAYSNNSIAIPT
234	H24	2070.4	GAENSVAYSNNSIAIPTNFT
235	H25	2112.5	NSVAYSNNSIAIPTNFTISV
236	H26	2143.5	AYSNNSIAIPTNFTISVTTE
237	H27	2145.6	NNSIAIPTNFTISVTTEILP
238	H28	2147.7	IAIPTNFTISVTTEILPVSM
239	H29	2180.6	PTNFTISVTTEILPVSMTKT
240	H30	2169.6	FTISVTTEILPVSMTKTSVD
241	I 1	2143.5	SVTTEILPVSMTKTSVDCTM
242	I 2	2235.7	TEILPVSMTKTSVDCTMYIC
243	I 3	2151.6	LPVSMTKTSVDCTMYICGDS
244	I 4	2175.5	SMTKTSVDCTMYICGDSTEC
245	I 5	2170.5	KTSVDCTMYICGDSTECSNL
246	I 6	2208.6	VDCTMYICGDSTECSNLLLQ
247	I 7	2198.7	TMYICGDSTECSNLLLQYGS
248	I 8	2154.6	ICGDSTECSNLLLQYGSFCT
249	I 9	2236.6	DSTECSNLLLQYGSFCTQLN
250	I10	2273.8	ECSNLLLQYGSFCTQLNRAL
251	I11	2225.9	NLLLQYGSFCTQLNRALTGI
252	I12	2184.7	LQYGSFCTQLNRALTGIAVE
**253**	**I13**	**2151.6**	**GSFCTQLNRALTGIAVEQDK**
**254**	**I14**	**2203.5**	**CTQLNRALTGIAVEQDKNTQ**
**255**	**I15**	**2246.6**	**LNRALTGIAVEQDKNTQEVF**
256	I16	2161.4	ALTGIAVEQDKNTQEVFAQV
**257**	**I17**	**2245.5**	**GIAVEQDKNTQEVFAQVKQI**
**258**	**I18**	**2396.6**	**VEQDKNTQEVFAQVKQIYKT**
**259**	**I19**	**2347.7**	**DKNTQEVFAQVKQIYKTPPI**
**260**	**I20**	**2380.8**	**TQEVFAQVKQIYKTPPIKDF**
**261**	**I21**	**2283.9**	**VFAQVKQIYKTPPIKDFGGF**
**262**	**I22**	**2314.9**	**QVKQIYKTPPIKDFGGFNFS**
**263**	**I23**	**2314**	**QIYKTPPIKDFGGFNFSQIL**
264	I24	2218.8	KTPPIKDFGGFNFSQILPDP
265	I25	2204.8	PIKDFGGFNFSQILPDPSKP
266	I26	2237.8	DFGGFNFSQILPDPSKPSKR
267	I27	2265.9	GFNFSQILPDPSKPSKRSFI
268	I28	2304.9	FSQILPDPSKPSKRSFIEDL
269	I29	2317	ILPDPSKPSKRSFIEDLLFN
270	I30	2321.9	DPSKPSKRSFIEDLLFNKVT
271	J 1	2322	KPSKRSFIEDLLFNKVTLAD
272	J 2	2285	KRSFIEDLLFNKVTLADAGF
273	J 3	2283	FIEDLLFNKVTLADAGFIKQ
274	J 4	2228.9	DLLFNKVTLADAGFIKQYGD
275	J 5	2160.8	FNKVTLADAGFIKQYGDCLG
276	J 6	2070.7	VTLADAGFIKQYGDCLGDIA
277	J 7	2099.7	ADAGFIKQYGDCLGDIAARD
278	J 8	2171.9	GFIKQYGDCLGDIAARDLIC
**279**	**J 9**	**2181.8**	**KQYGDCLGDIAARDLICAQK**
**280**	**J10**	**2080.7**	**GDCLGDIAARDLICAQKFNG**
**281**	**J11**	**2118.8**	**LGDIAARDLICAQKFNGLTV**
**282**	**J12**	**2140.8**	**IAARDLICAQKFNGLTVLPP**
283	J13	2212.9	RDLICAQKFNGLTVLPPLLT
284	J14	2203.8	ICAQKFNGLTVLPPLLTDEM
285	J15	2228.8	QKFNGLTVLPPLLTDEMIAQ
286	J16	2176.7	NGLTVLPPLLTDEMIAQYTS
287	J17	2189.8	TVLPPLLTDEMIAQYTSALL
288	J18	2105.7	PPLLTDEMIAQYTSALLAGT
289	J19	2099.7	LTDEMIAQYTSALLAGTITS
290	J20	2114.7	EMIAQYTSALLAGTITSGWT
291	J21	2016.6	AQYTSALLAGTITSGWTFGA
292	J22	1853.5	TSALLAGTITSGWTFGAGAA
293	J23	1948.7	LLAGTITSGWTFGAGAALQI
294	J24	1966.6	GTITSGWTFGAGAALQIPFA
295	J25	2085.7	TSGWTFGAGAALQIPFAMQM
296	J26	2230.9	WTFGAGAALQIPFAMQMAYR
297	J27	2114.8	GAGAALQIPFAMQMAYRFNG
298	J28	2198.9	AALQIPFAMQMAYRFNGIGV
299	J29	2286.8	QIPFAMQMAYRFNGIGVTQN
300	J30	2323.9	FAMQMAYRFNGIGVTQNVLY
301	K 1	2345.7	QMAYRFNGIGVTQNVLYENQ
**302**	**K 2**	**2369.9**	**YRFNGIGVTQNVLYENQKLI**
**303**	**K 3**	**2216.6**	**NGIGVTQNVLYENQKLIANQ**
**304**	**K 4**	**2280.6**	**GVTQNVLYENQKLIANQFNS**
**305**	**K 5**	**2264.7**	**QNVLYENQKLIANQFNSAIG**
**306**	**K 6**	**2292.9**	**LYENQKLIANQFNSAIGKIQ**
**307**	**K 7**	**2202.8**	**NQKLIANQFNSAIGKIQDSL**
308	K 8	2107.7	LIANQFNSAIGKIQDSLSST
309	K 9	2039.5	NQFNSAIGKIQDSLSSTASA
310	K10	1948.6	NSAIGKIQDSLSSTASALGK
311	K11	2032.7	IGKIQDSLSSTASALGKLQD
312	K12	2046.5	IQDSLSSTASALGKLQDVVN
313	K13	2003.4	SLSSTASALGKLQDVVNQNA
314	K14	2028.4	STASALGKLQDVVNQNAQAL
315	K15	2097.5	SALGKLQDVVNQNAQALNTL
**316**	**K16**	**2181.5**	**GKLQDVVNQNAQALNTLVKQ**
**317**	**K17**	**2170.4**	**QDVVNQNAQALNTLVKQLSS**
**318**	**K18**	**2146.5**	**VNQNAQALNTLVKQLSSNFG**
**319**	**K19**	**2076.6**	**NAQALNTLVKQLSSNFGAIS**
320	K20	2062.7	ALNTLVKQLSSNFGAISSVL
321	K21	2106.7	TLVKQLSSNFGAISSVLNDI
322	K22	2149.8	KQLSSNFGAISSVLNDILSR
323	K23	2136.8	SSNFGAISSVLNDILSRLDK
324	K24	2147.8	FGAISSVLNDILSRLDKVEA
325	K25	2228.7	ISSVLNDILSRLDKVEAEVQ
326	K26	2325.8	VLNDILSRLDKVEAEVQIDR
327	K27	2326.9	DILSRLDKVEAEVQIDRLIT
328	K28	2311.9	SRLDKVEAEVQIDRLITGRL
329	K29	2283.8	DKVEAEVQIDRLITGRLQSL
330	K30	2333.8	EAEVQIDRLITGRLQSLQTY
331	L 1	2332.8	VQIDRLITGRLQSLQTYVTQ
332	L 2	2346.9	DRLITGRLQSLQTYVTQQLI
333	L 3	2260.8	ITGRLQSLQTYVTQQLIRAA
334	L 4	2387.9	RLQSLQTYVTQQLIRAAEIR
335	L 5	2219.7	SLQTYVTQQLIRAAEIRASA
336	L 6	2189.7	TYVTQQLIRAAEIRASANLA
337	L 7	2126.7	TQQLIRAAEIRASANLAATK
338	L 8	2116.8	LIRAAEIRASANLAATKMSE
339	L 9	2049.6	AAEIRASANLAATKMSECVL
340	L10	2050.6	IRASANLAATKMSECVLGQS
341	L11	2093.6	SANLAATKMSECVLGQSKRV
342	L12	2186.7	LAATKMSECVLGQSKRVDFC
**343**	**L13**	**2173.7**	**TKMSECVLGQSKRVDFCGKG**
**344**	**L14**	**2226.7**	**SECVLGQSKRVDFCGKGYHL**
**345**	**L15**	**2272.9**	**VLGQSKRVDFCGKGYHLMSF**
**346**	**L16**	**2315.8**	**QSKRVDFCGKGYHLMSFPQS**
347	L17	2277.7	RVDFCGKGYHLMSFPQSAPH
348	L18	2162.6	FCGKGYHLMSFPQSAPHGVV
349	L19	2252.7	KGYHLMSFPQSAPHGVVFLH
350	L20	2267.6	HLMSFPQSAPHGVVFLHVTY
351	L21	2153.4	SFPQSAPHGVVFLHVTYVPA
**352**	**L22**	**2207.4**	**QSAPHGVVFLHVTYVPAQEK**
**353**	**L23**	**2283.5**	**PHGVVFLHVTYVPAQEKNFT**

**354**	**L24**	**2261.5**	**VVFLHVTYVPAQEKNFTTAP**
355	L25	2203.5	LHVTYVPAQEKNFTTAPAIC
356	L26	2163.4	TYVPAQEKNFTTAPAICHDG
357	L27	2136.4	PAQEKNFTTAPAICHDGKAH
358	L28	2240.6	EKNFTTAPAICHDGKAHFPR
359	L29	2154.5	FTTAPAICHDGKAHFPREGV
360	L30	2138.5	APAICHDGKAHFPREGVFVS
361	M 1	2171.5	ICHDGKAHFPREGVFVSNGT
362	M 2	2288.6	DGKAHFPREGVFVSNGTHWF
363	M 3	2316.5	AHFPREGVFVSNGTHWFVTQ
364	M 4	2378.6	PREGVFVSNGTHWFVTQRNF
365	M 5	2385.6	GVFVSNGTHWFVTQRNFYEP
366	M 6	2436.7	VSNGTHWFVTQRNFYEPQII
367	M 7	2453.7	GTHWFVTQRNFYEPQIITTD
368	M 8	2520.8	WFVTQRNFYEPQIITTDNTF
369	M 9	2331.6	TQRNFYEPQIITTDNTFVSG
370	M10	2278.5	NFYEPQIITTDNTFVSGNCD
371	M11	2165.4	EPQIITTDNTFVSGNCDVVI
372	M12	2080.5	IITTDNTFVSGNCDVVIGIV
373	M13	2082.3	TDNTFVSGNCDVVIGIVNNT
374	M14	2129.4	TFVSGNCDVVIGIVNNTVYD
375	M15	2120.4	SGNCDVVIGIVNNTVYDPLQ
376	M16	2201.5	CDVVIGIVNNTVYDPLQPEL
377	M17	2233.6	VIGIVNNTVYDPLQPELDSF
378	M18	2350.6	IVNNTVYDPLQPELDSFKEE
**379**	**M19**	**2380.7**	**NTVYDPLQPELDSFKEELDK**
**380**	**M20**	**2505**	**YDPLQPELDSFKEELDKYFK**
**381**	**M21**	**2481.9**	**LQPELDSFKEELDKYFKNHT**
382	M22	2442.8	ELDSFKEELDKYFKNHTSPD
383	M23	2412.8	SFKEELDKYFKNHTSPDVDL
384	M24	2335.7	EELDKYFKNHTSPDVDLGDI
385	M25	2221.7	DKYFKNHTSPDVDLGDISGI
386	M26	2087.5	FKNHTSPDVDLGDISGINAS
387	M27	2010.3	HTSPDVDLGDISGINASVVN
388	M28	2054.5	PDVDLGDISGINASVVNIQK
389	M29	2100.6	DLGDISGINASVVNIQKEID
390	M30	2198.7	DISGINASVVNIQKEIDRLN
391	N 1	2182.6	GINASVVNIQKEIDRLNEVA
392	N 2	2253.7	ASVVNIQKEIDRLNEVAKNL
393	N 3	2326.7	VNIQKEIDRLNEVAKNLNES
394	N 4	2341.8	QKEIDRLNEVAKNLNESLID
395	N 5	2326.8	IDRLNEVAKNLNESLIDLQE
**396**	**N 6**	**2240.8**	**LNEVAKNLNESLIDLQELGK**
**397**	**N 7**	**2304.8**	**VAKNLNESLIDLQELGKYEQ**
**398**	**N 8**	**2411**	**NLNESLIDLQELGKYEQYIK**
399	N 9	2539.1	ESLIDLQELGKYEQYIKWPW
400	N10	2672.3	IDLQELGKYEQYIKWPWYIW
401	N11	2648.3	QELGKYEQYIKWPWYIWLGF
402	N12	2519.3	GKYEQYIKWPWYIWLGFIAG
403	N13	2468.3	EQYIKWPWYIWLGFIAGLIA
404	N14	2391.4	IKWPWYIWLGFIAGLIAIVM
405	N15	2277.2	PWYIWLGFIAGLIAIVMVTI
406	N16	2178.2	IWLGFIAGLIAIVMVTIMLC
407	N17	2101	GFIAGLIAIVMVTIMLCCMT
408	N18	2076.8	AGLIAIVMVTIMLCCMTSCC
409	N19	2138.8	IAIVMVTIMLCCMTSCCSCL
410	N20	2129.7	VMVTIMLCCMTSCCSCLKGC
411	N21	2093.6	TIMLCCMTSCCSCLKGCCSC
412	N22	1995.4	LCCMTSCCSCLKGCCSCGSC
413	N23	2054.5	MTSCCSCLKGCCSCGSCCKF
414	N24	2094.4	CCSCLKGCCSCGSCCKFDED
415	N25	2132.4	CLKGCCSCGSCCKFDEDDSE
416	N26	2097.3	GCCSCGSCCKFDEDDSEPVL
417	N27	2118.4	SCGSCCKFDEDDSEPVLKGV
418	N28	2249.6	SCCKFDEDDSEPVLKGVKLH
419	N29	2323.7	CKFDEDDSEPVLKGVKLHYT
420	N30	0	
	(newline)		

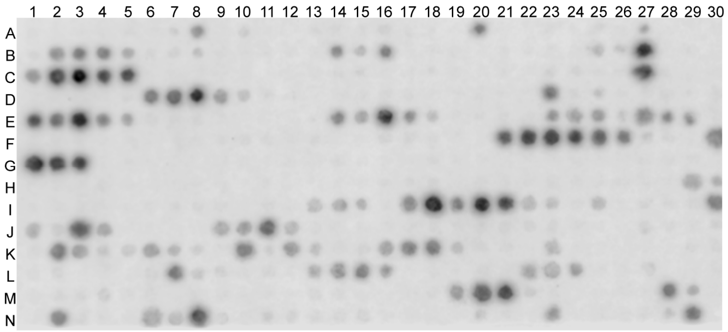

SPOT: Vimentin DETECT: IR800/S1S2ECD

421	O 1	2151.7	MSTRSVSSSSYRRMFGGPGT
422	O 2	2146.7	RSVSSSSYRRMFGGPGTASR
423	O 3	2075.6	SSSSYRRMFGGPGTASRPSS
424	O 4	2144.7	SYRRMFGGPGTASRPSSSRS
425	O 5	2101.6	RMFGGPGTASRPSSSRSYVT
426	O 6	1956.3	GGPGTASRPSSSRSYVTTST
427	O 7	2165.5	GTASRPSSSRSYVTTSTRTY
428	O 8	2193.6	SRPSSSRSYVTTSTRTYSLG
429	O 9	2124.6	SSSRSYVTTSTRTYSLGSAL
430	O10	2203.7	RSYVTTSTRTYSLGSALRPS
431	O11	2141.6	VTTSTRTYSLGSALRPSTSR
432	O12	2203.8	STRTYSLGSALRPSTSRSLY
433	O13	2104.7	TYSLGSALRPSTSRSLYASS
434	O14	1964.6	LGSALRPSTSRSLYASSPGG
435	O15	2040.6	ALRPSTSRSLYASSPGGVYA
436	O16	2044.5	PSTSRSLYASSPGGVYATRS
437	O17	2016.5	SRSLYASSPGGVYATRSSAV
438	O18	2111.7	LYASSPGGVYATRSSAVRLR
439	O19	2037.5	SSPGGVYATRSSAVRLRSSV
440	O20	2019.5	GGVYATRSSAVRLRSSVPGV
441	O21	2188.8	YATRSSAVRLRSSVPGVRLL
442	O22	2183.7	RSSAVRLRSSVPGVRLLQDS
443	O23	2214.7	AVRLRSSVPGVRLLQDSVDF
444	O24	2159.7	LRSSVPGVRLLQDSVDFSLA
445	O25	2102.6	SVPGVRLLQDSVDFSLADAI
446	O26	2163.6	GVRLLQDSVDFSLADAINTE
447	O27	2240.7	LLQDSVDFSLADAINTEFKN
448	O28	2244.6	DSVDFSLADAINTEFKNTRT
449	O29	2314.7	DFSLADAINTEFKNTRTNEK
450	O30	2306.7	LADAINTEFKNTRTNEKVEL
451	P 1	2377.7	AINTEFKNTRTNEKVELQEL
452	P 2	2464.7	TEFKNTRTNEKVELQELNDR
453	P 3	2419.7	KNTRTNEKVELQELNDRFAN
454	P 4	2467.8	RTNEKVELQELNDRFANYID
455	P 5	2479.9	EKVELQELNDRFANYIDKVR
456	P 6	2513	ELQELNDRFANYIDKVRFLE
457	P 7	2512.9	ELNDRFANYIDKVRFLEQQN
458	P 8	2511.1	DRFANYIDKVRFLEQQNKIL
459	P 9	2406	ANYIDKVRFLEQQNKILLAE
460	P10	2428	IDKVRFLEQQNKILLAELEQ
461	P11	2370	VRFLEQQNKILLAELEQLKG
462	P12	2280.9	LEQQNKILLAELEQLKGQGK
463	P13	2267	QNKILLAELEQLKGQGKSRL
464	P14	2182	ILLAELEQLKGQGKSRLGDL
465	P15	2263.8	AELEQLKGQGKSRLGDLYEE
466	P16	2366.9	EQLKGQGKSRLGDLYEEEMR
467	P17	2395	KGQGKSRLGDLYEEEMRELR
468	P18	2465	GKSRLGDLYEEEMRELRRQV
469	P19	2549	RLGDLYEEEMRELRRQVDQL
470	P20	2552.8	DLYEEEMRELRRQVDQLTND
471	P21	2516.8	EEEMRELRRQVDQLTNDKAR
472	P22	2456.8	MRELRRQVDQLTNDKARVEV
473	P23	2440.7	LRRQVDQLTNDKARVEVERD
474	P24	2313.5	QVDQLTNDKARVEVERDNLA
475	P25	2328.6	QLTNDKARVEVERDNLAEDI
476	P26	2386.8	NDKARVEVERDNLAEDIMRL
477	P27	2442.9	ARVEVERDNLAEDIMRLREK
478	P28	2486.9	EVERDNLAEDIMRLREKLQE
479	P29	2503.1	RDNLAEDIMRLREKLQEEML
480	P30	2531.1	LAEDIMRLREKLQEEMLQRE
481	Q 1	2547	DIMRLREKLQEEMLQREEAE
482	Q 2	2515.9	RLREKLQEEMLQREEAENTL
483	Q 3	2452.7	EKLQEEMLQREEAENTLQSF
484	Q 4	2481.6	QEEMLQREEAENTLQSFRQD
485	Q 5	2423.6	MLQREEAENTLQSFRQDVDN
486	Q 6	2322.5	REEAENTLQSFRQDVDNASL
487	Q 7	2248.6	AENTLQSFRQDVDNASLARL
488	Q 8	2291.7	TLQSFRQDVDNASLARLDLE
489	Q 9	2332.8	SFRQDVDNASLARLDLERKV
490	Q10	2271.7	QDVDNASLARLDLERKVESL
491	Q11	2315.7	DNASLARLDLERKVESLQEE
492	Q12	2346.9	SLARLDLERKVESLQEEIAF
493	Q13	2445.1	RLDLERKVESLQEEIAFLKK
494	Q14	2440	LERKVESLQEEIAFLKKLHE
495	Q15	2412.9	KVESLQEEIAFLKKLHEEEI
496	Q16	2426.9	SLQEEIAFLKKLHEEEIQEL
497	Q17	2425.8	EEIAFLKKLHEEEIQELQAQ
498	Q18	2424.8	AFLKKLHEEEIQELQAQIQE
499	Q19	2457.6	KKLHEEEIQELQAQIQEQHV
500	Q20	2444.4	HEEEIQELQAQIQEQHVQID
501	Q21	2362.4	EIQELQAQIQEQHVQIDVDV
502	Q22	2304.4	ELQAQIQEQHVQIDVDVSKP
503	Q23	2263.4	AQIQEQHVQIDVDVSKPDLT
504	Q24	2206.4	QEQHVQIDVDVSKPDLTAAL
505	Q25	2191.5	HVQIDVDVSKPDLTAALRDV
506	Q26	2239.6	IDVDVSKPDLTAALRDVRQQ
507	Q27	2291.6	DVSKPDLTAALRDVRQQYES
508	Q28	2231.6	KPDLTAALRDVRQQYESVAA
509	Q29	2246.7	LTAALRDVRQQYESVAAKNL
510	Q30	2289.6	ALRDVRQQYESVAAKNLQEA
511	R 1	2393.5	DVRQQYESVAAKNLQEAEEW
512	R 2	2401.6	QQYESVAAKNLQEAEEWYKS
**513**	**R 3**	**2328.7**	**ESVAAKNLQEAEEWYKSKFA**
**514**	**R 4**	**2328.8**	**AAKNLQEAEEWYKSKFADLS**
**515**	**R 5**	**2329.7**	**NLQEAEEWYKSKFADLSEAA**
516	R 6	2358.7	EAEEWYKSKFADLSEAANRN
517	R 7	2329.7	EWYKSKFADLSEAANRNNDA
518	R 8	2248.7	KSKFADLSEAANRNNDALRQ
519	R 9	2232.6	FADLSEAANRNNDALRQAKQ
520	R10	2216.5	LSEAANRNNDALRQAKQEST
521	R11	2335.6	AANRNNDALRQAKQESTEYR
522	R12	2462.7	RNNDALRQAKQESTEYRRQV
523	R13	2406.7	DALRQAKQESTEYRRQVQSL
524	R14	2440.6	RQAKQESTEYRRQVQSLTCE
525	R15	2370.5	KQESTEYRRQVQSLTCEVDA
526	R16	2283.6	STEYRRQVQSLTCEVDALKG
527	R17	2310.6	YRRQVQSLTCEVDALKGTNE
528	R18	2164.4	QVQSLTCEVDALKGTNESLE
529	R19	2224.6	SLTCEVDALKGTNESLERQM
530	R20	2339.7	CEVDALKGTNESLERQMREM
531	R21	2380.7	DALKGTNESLERQMREMEEN
532	R22	2398.7	KGTNESLERQMREMEENFAV
533	R23	2383.6	NESLERQMREMEENFAVEAA
534	R24	2458.7	LERQMREMEENFAVEAANYQ
535	R25	2389.6	QMREMEENFAVEAANYQDTI
536	R26	2300.6	EMEENFAVEAANYQDTIGRL
537	R27	2283.5	ENFAVEAANYQDTIGRLQDE
538	R28	2248.5	AVEAANYQDTIGRLQDEIQN
539	R29	2337.7	AANYQDTIGRLQDEIQNMKE
540	R30	2412.8	YQDTIGRLQDEIQNMKEEMA
541	S 1	2412.9	TIGRLQDEIQNMKEEMARHL
542	S 2	2590	RLQDEIQNMKEEMARHLREY
543	S 3	2548.9	DEIQNMKEEMARHLREYQDL
544	S 4	2517.9	QNMKEEMARHLREYQDLLNV
545	S 5	2475	KEEMARHLREYQDLLNVKMA
546	S 6	2430.1	MARHLREYQDLLNVKMALDI
547	S 7	2385	HLREYQDLLNVKMALDIEIA
548	S 8	2399	EYQDLLNVKMALDIEIATYR
549	S 9	2333.2	DLLNVKMALDIEIATYRKLL
550	S10	2307	NVKMALDIEIATYRKLLEGE
551	S11	2338	MALDIEIATYRKLLEGEESR
552	S12	2336	DIEIATYRKLLEGEESRISL
553	S13	2286	IATYRKLLEGEESRISLPLP
554	S14	2349	YRKLLEGEESRISLPLPNFS
555	S15	2215.8	LLEGEESRISLPLPNFSSLN
556	S16	2258.8	GEESRISLPLPNFSSLNLRE
557	S17	2271.9	SRISLPLPNFSSLNLRETNL
558	S18	2230.8	SLPLPNFSSLNLRETNLDSL
559	S19	2242.8	LPNFSSLNLRETNLDSLPLV
560	S20	2271.7	FSSLNLRETNLDSLPLVDTH
561	S21	2321.8	LNLRETNLDSLPLVDTHSKR
562	S22	2308.8	RETNLDSLPLVDTHSKRTLL
563	S23	2264.9	NLDSLPLVDTHSKRTLLIKT
564	S24	2251.8	SLPLVDTHSKRTLLIKTVET
565	S25	2282.8	LVDTHSKRTLLIKTVETRDG
566	S26	2295.8	THSKRTLLIKTVETRDGQVI
567	S27	2314.8	KRTLLIKTVETRDGQVINET
568	S28	2281.6	LLIKTVETRDGQVINETSQH
569	S29	2309.3	KTVETRDGQVINETSQHHDD
570	S30	2322.3	VETRDGQVINETSQHHDDLE

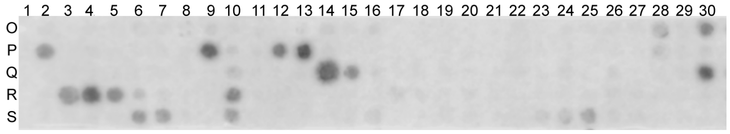

Supplement: Supplementary file 1 [file ijms-25-02477-s001.zip › Supplemental Table 1-SPOT Membrane Map and binding.pdf]
